# Bioinformatics analysis to screen for genes related to myocardial infarction

**DOI:** 10.3389/fgene.2022.990888

**Published:** 2022-10-10

**Authors:** Liting Yang, Xuyang Pan, Ying Zhang, Dongsheng Zhao, Liang Wang, Guoliang Yuan, Changgao Zhou, Tao Li, Wei Li

**Affiliations:** Department of Cardiology, Shuyang Hospital of Traditional Chinese Medicine, Suqian, Jiangsu, China

**Keywords:** myocardial infarction, differentially expressed genes, weighed gene co-expression network analysis, gene co-expression network, support vector machine

## Abstract

Myocardial infarction (MI) is an acute and persistent myocardial ischemia caused by coronary artery disease. This study screened potential genes related to MI. Three gene expression datasets related to MI were downloaded from the Gene Expression Omnibus database. Differentially expressed genes (DEGs) were screened using the MetaDE package. Afterward, the modules and genes closely related to MI were screened and a gene co-expression network was constructed. A support vector machine (SVM) classification model was then constructed based on the GSE61145 dataset using the e1071 package in R. A total of 98 DEGs were identified in the MI samples. Next, three modules associated with MI were screened and an SVM classification model involving seven genes was constructed. Among them, *BCL6, CEACAM8*, and *CUGBP2* showed co-interactions in the gene co-expression network. Therefore, *ACOX1, BCL6, CEACAM8,* and *CUGBP2*, in addition to *GPX7*, might be feature genes related to MI.

## Introduction

Myocardial infarction (MI), a major cause of death and disability worldwide, is caused by myocardial cell death due to prolonged ischemia (Thygesen et al., 2007). The most important risk factors of MI include age, smoking, hypertension, diabetes, and total and high-density lipoprotein cholesterol levels (Bao et al., 2022; Bruyninckx et al., 2008;O’Gara, 2013). Chest pain is the most common clinical manifestation of acute MI, which is often described as stress or compression (Fauci, 2014). The pain often radiates to the left arm as well as to the jaw, neck, right arm, back, and upper abdomen (Marcus et al., 2007). Approximately 15.9 million people worldwide developed MI in 2015 (Ji et al., 2015; Cowan et al., 2018). MI is an emerging public health concern globally. Previous studies have suggested that *ALOX5AP* (arachidonate 5-lipoxygenase activating protein) confers a risk of MI; thus, *ALOX5AP* is the first specific gene conferring a substantial population-attributable risk (PAR) of MI (Helgadottir et al., 2004). *TGF-β1* (Transforming growth factor-beta 1) is involved in the modulation of cell growth and differentiation, and plays an important role in cardiovascular physiopathology and the repair of vascular injury (Nikol et al., 1992; Cambien et al., 1996). Meanwhile, the *ALDH2* (aldehyde dehydrogenase 2) *Lys/Lys* genotype is a risk factor for MI due to its influence on high-density lipoprotein (HDL) cholesterol level (Gardemann et al., 1998; Takagi et al., 2002). *PLA1* (Phospholipase A1 member A) hydrolyzes fatty acids at the sn-1 position of phosphatidylserine and 1-acyl-2-lysophosphatidylserine and its abnormal expression is associated with coronary artery disease (CAD) and MI (Ji et al., 2019). Furthermore, high-throughput screening revealed that Nox2 as a potential miRNA target for function improvement following MI (Wang et al., 2012; Smyth and Smyth, 2013; Yang et al., 2017; Bao et al., 2022; Kim et al., 2022). However, the genes closely related to MI development have not been fully identified.

The present study searched microarray datasets related to human MI. Three gene expression datasets on MI were downloaded from the Gene Expression Omnibus database and differentially expressed genes (DEGs) were identified using MetaDE. The genes associated with MI were further screened by identifying disease-associated modules. With this information, we constructed a gene co-expression network. To classify the MI samples, a support vector machine (SVM) classification model trained on the GSE61145 dataset was used. With this trained model, we focused on mining related genes associated with MI.

## Methods

### Microarray data

The GSE61145, GSE60993, and GSE34198 gene expression datasets related to human MI, which were developed based on the GPL6106, GPL6884, and GPL6102 platforms, respectively, were downloaded from the Gene Expression Omnibus (GEO, http://www.ncbi.nlm.nih.gov/geo/) database. The GSE61145 dataset contained data on 14 blood samples from patients with MI and 10 samples from normal controls. The GSE60993 dataset included data on a total of 24 samples (7 and 17 blood samples from normal controls and patients with MI, respectively). Finally, the GSE34198 dataset contained 97 samples (48 and 49 blood samples from normal controls and patients with MI, respectively).

The raw data were downloaded and the probes were annotated into gene symbols based on platform annotation information. Because a single gene could correspond to several probes (multiple values), the average gene expression values were calculated for each gene. Afterward, log2 conversion was performed to transform the gene expression data from a skewed distribution to an approximately normal distribution. The data were then normalized using the limma package (MetaDE) (http://www.bioconductor.org/packages/2.9/bioc/html/limma.html) in R language.

### Differentially expressed gene and meta-analyses

DEGs were screened by using the MetaDE package (Wang et al., 2012) in R based on the GSE61145 and GSE60993 datasets. The raw data were downloaded and the probes were annotated into gene symbols based on platform annotation information. The average gene expression values were calculated for each gene. Afterward, log2 conversion was performed to transform the gene expression data from a skewed distribution to an approximately normal distribution. The data were then normalized using the limma package in R language. The heterogeneity of gene expression data based on different platforms was analyzed using the MetaDE.ES method (Kim et al., 2022), with tau^2^ = 0 and Qpval >0.05. Differential expression analysis of genes with homogeneous expression was then performed between the disease and control groups, with an FDR (false discovery rate) of < 0.05 defined as the threshold value.

### Screening modules and disease-related genes based on the meta-analysis

Weighted gene co-expression network analysis (WGCNA) (MetaDE) is a typical system biology algorithm used to construct gene co-expression networks based on high-throughput mRNA expression data. The genes and modules related to MI in this study were analyzed for DEGs based on the WGCNA algorithm (Langfelder and Horvath, 2008). The correlation coefficient between gene expression was calculated using the function $S_{mn}=\left|{cor}_{\left(m,n\right)}\right|$ . Then, the coefficient was then weighted by the exponential adjacency function $a_{mn}={power}_{\left(S_{mn},\beta \right)}$ . According to the principle of scale-free networks, the weight coefficient β was determined for the adjacency function. To measure the dissimilarity between nodes, the correlation matrix (S_mn_) was transformed into an adjacency matrix (a_mn_). A hierarchical clustering tree was then constructed based on the dissimilarity coefficients between genes, with different branches of the clustering tree representing different gene modules. Finally, *t*-tests were used to analyze the correlations between network modules and disease states.

### Construction of the gene co-expression network and enrichment analysis

The co-expressed modules that were closely associated with disease state were analyzed and the module genes were collected to construct the co-expression network. Genes related to disease were subjected to gene ontology (GO) enrichment analysis using the clusterProfiler package (Yu et al., 2012) in R based on the hypergeometric distribution algorithm. *P* < 0.05 was defined as the threshold value. The formula for the hypergeometric distribution algorithm is shown in Eq. 1.

$$p=1-\overset{H-1}{\underset{i=0}{\sum }}\frac{\left(\begin{array}{l} M\\ i \end{array}\right)\left(\begin{array}{l} N-M\\ K-H \end{array}\right)}{\left(\begin{array}{l} N\\ K \end{array}\right)},$$ (1)

where N represents the number of genes with GO functional annotations; K represents the number of DEGs among N genes, and M represents the number of genes that are annotated with a particular GO function.

### Support vector machine (SVM) classification modeling

To classify the samples, an optimal classification hyperplane must be selected from numerous options to maximize the distance δ between the sample set and the classification hyperplane. When ε = |*wx*_*i*_ + *b*| = 1, the distance between the two types of sample points is 2 ((|*wx*_*i*_ + *b*|)/‖*w*‖) =(2/‖*w*‖). The goal is to construct an optimal classification hyperplane under the constraint of Eq. 2 to maximize (2/‖*w*‖) and minimize (‖*w*‖2/2).

$$\begin{array}{l} \left\{ \begin{array}{l} wx_{i}+b\geq 1,y_{i}=1\\ wx_{i}+b\leq -1,y_{i}=-1 \end{array}\right.\\ i=1,2\cdots ,l \end{array}.$$ (2)

Most classification issues can be treated as nonlinear separable problems, and quadratic programming problems can be modified as follows by introducing the slack variable *ξi* in Eq. 3.

$$\begin{array}{l} \left\{ \begin{array}{l} \min\frac{1}{2}{\left\|w\right\|}^{2}+C\sum \xi_{i},\xi_{i}\geq 0\\ {\text{constraint}\;\text{condition}:\;y}_{i}\left(\left(wx_{i}+b\right)\right)\geq 1-\xi_{i} \end{array}\right.,\\ i=1,2\cdots ,l \end{array}$$ (3)

where *ξ*_*i*_ is the slack variable and *C* is the penalty coefficient.

With GSE61145 as the training dataset and all genes of interest as classification factors, the SVM model was established using the e1071 package (MetaDE) in Rto distinguish the disease and control samples. The classification factors were added individually until all of them had been added to the SVM classifier. The classification accuracy of the SVM classifier was then calculated and the genes that affected classification accuracy were removed. The SVM model was then validated in the GSE60993 and GSE34198 gene expression datasets.

## Results

### DEG screening

A total of 1,231 DEGs were identified by the MetaDE package (Langfelder and Horvath, 2008; Wang et al., 2012; Yu et al., 2012; Meyer, 2013). The top 10 DEGs are listed in Table 1, including *GZMK* (granzyme K), *HLA-DQA* (histocompatibility complex, class II, DQ alpha), and *EOMES* (eomesodermin). First, the heterogeneity of gene expression data based on different platforms was analyzed using the MetaDE.ES method, with tau2 = 0 and Qpval >0.05. Then, the differential expression analysis of genes with homogeneous expression was conducted between the disease and control groups, with an FDR (false discovery rate) of <0.05 defined as the threshold value. A total of 1,231 DEGs were identified. The top 10 DEGs with the smallest *p*-values in the gene difference analysis between the disease and control groups were selected; that is, the genes with the largest difference between disease and control groups. The present study analyzed the co-expressed modules that were closely associated with the disease state and identified the module genes to construct a co-expression network. Genes related to disease were subjected to gene ontology (GO) enrichment analysis. KEGG pathway enrichment analysis was not performed.

**TABLE 1:** List of top 10 significant differentially expressed genes from GSE61145 and GSE60993.

Symbol	*p*	FDR	Q	Qp	tau2	logFC
GZMK	1.00E-20	2.47E-17	0.661264	0.416114	0	−4.38757
HLA-DQA1	1.22E-06	0.00028	0.067206	0.795449	0	−3.34521
EOMES	1.22E-06	0.00028	0.02484	0.874767	0	−3.27466
GZMA	8.51E-06	0.000625	0.599134	0.438909	0	−3.22898
GZMH	4.62E-05	0.001564	0.05081	0.82166	0	−2.76322
GZMM	5.67E-06	0.000478	0.164991	0.684602	0	−2.74506
KLRB1	4.05E-07	0.000133	0.336467	0.561876	0	−2.74359
NKG7	1.09E-05	0.00069	0.001251	0.971784	0	−2.718
IL2RB	1.38E-05	0.000778	0.644833	0.421966	0	−2.62686

a FDR, false discovery rate; FC, fold-change.

### Modules and genes closely related to disease

**FIGURE 1:**
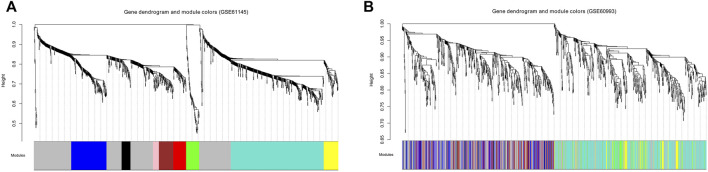
Tree diagrams for identifying the disease-associated modules based on the GSE61145 **(A)** and GSE60993 **(B)** datasets. The abscissa represents modules in different colors. The ordinate represents the height of the system clustering tree based on the expression value.

**FIGURE 2:**
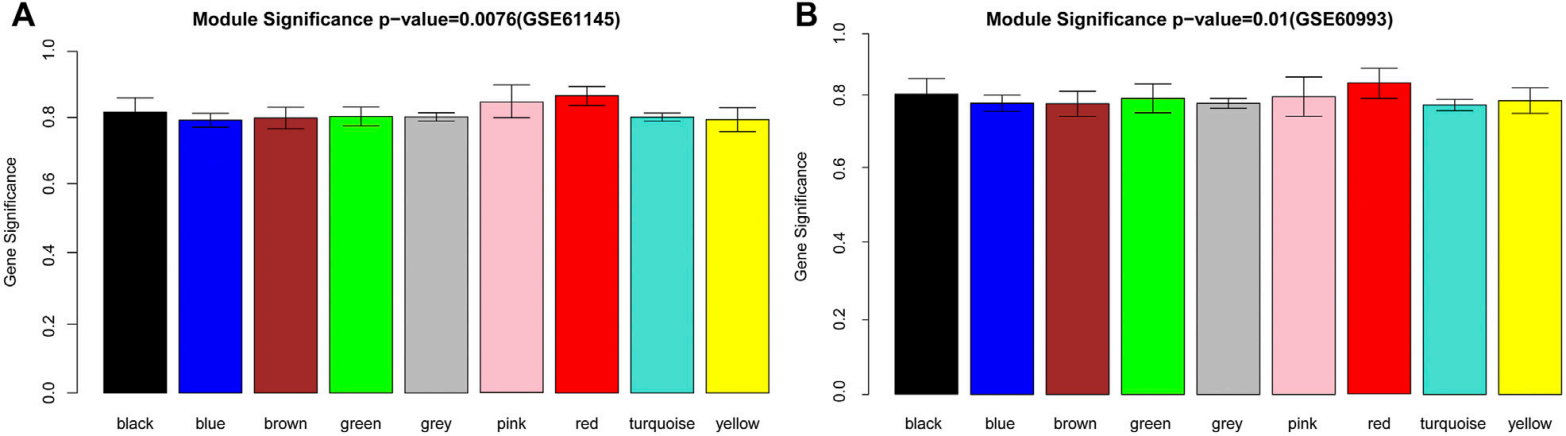
The disease-associated modules identified from the GSE61145 **(A)** and GSE60993 **(B)** datasets. The abscissa represents modules in different colors. The ordinate represents the overall correlation coefficient between the genes in each module and the disease state.

To satisfy the precondition of scale-free network distribution, we selected a power of 18 as the adjacency parameter. The results of the consistency analysis showed a high correlation between the GSE61145 and GSE60993 datasets (correlation coefficient = 0.86, *p*-value < 1e-200). Additionally, GSE61145 was used as a training set to identify disease-associated modules (Figure 1A). Module partitioning for the GSE60993 dataset (Figure 1B) showed high consistency with the GSE61145 dataset. We then calculated the correlation coefficient between module and disease state (normal and MI samples) for the GSE61145 (Figure 2A) and GSE60993 (Figure 2B) datasets, respectively (Table 1). According to the correlation coefficients, the top three modules (black, pink, and red) were identified.

### Gene co-expression network construction and enrichment analysis

**FIGURE 3:**
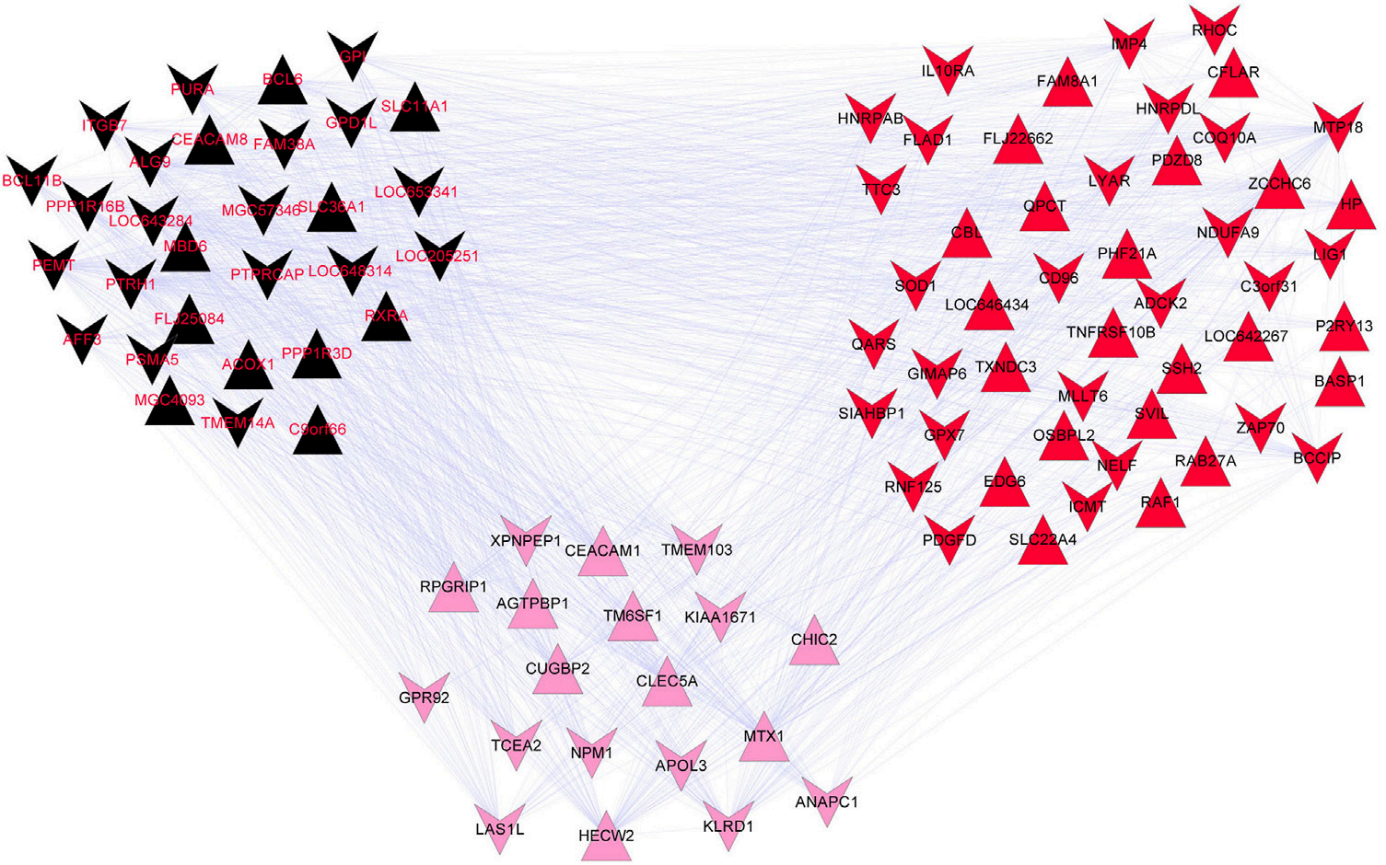
Gene co-expression network of the black, pink, and red modules. The inverted and positive triangles represent up- and down-regulated genes in the disease group, respectively. The node colors reflects the colors of the disease modules.

The correlation coefficients between genes in the top three modules and disease state were calculated, which revealed 98 genes with correlation coefficients >0.5. These included 30 genes (11 up-regulated and 19 down-regulated) in the black module, 19 genes (9 up-regulated and 10 down-regulated) in the pink module, and 49 genes (22 up-regulated and 27 down-regulated) in the red module. The gene co-expressed networks of the 98 genes were then constructed (Figure 3). GO analysis showed the enrichment of 10 GO terms among the genes in the black module (Table 2) and 15 GO terms among the genes in the red module (Table 2). The GO terms enriched in the black module included negative regulation of cell proliferation (*p*-value = 0.009704), regulation of cell proliferation (*p*-value = 0.014724), and positive regulation of macromolecule metabolic process (*p*-value = 0.019608). The GO terms closely related to the genes in the red module mainly included positive regulation of I-kappaB kinase/NF-kappaB cascade (*p*-value = 0.024598), regulation of I-kappaB kinase/NF-kappaB cascade (*p*-value = 0.029497), and positive regulation of signal transduction (*p*-value = 0.037345). No GO terms were significantly enriched among the genes in the pink module.

**TABLE 2:** Gene ontology functions enriched in the black (A) and red (B) modules.

Term	Count	*p*-value	Genes
(A)			
GO:0008285∼negative regulation of cell proliferation	4	0.009704	BCL11B, RXRA, PEMT, BCL6
GO:0042127∼regulation of cell proliferation	5	0.014724	BCL11B, RXRA, PEMT, BCL6, PURA
GO:0010604∼positive regulation of macromolecule metabolic process	5	0.019608	SLC11A1, PSMA5, BCL11B, RXRA, PEMT
GO:0046649∼lymphocyte activation	3	0.025327	SLC11A1, BCL11B, BCL6
GO:0019637∼organophosphate metabolic process	3	0.025564	GPD1L, PEMT, ALG9
GO:0015807∼L-amino acid transport	2	0.02853	SLC36A1, SLC11A1
GO:0045321∼leukocyte activation	3	0.036326	SLC11A1, BCL11B, BCL6
GO:0000060∼protein import into nucleus, translocation	2	0.041902	SLC11A1, BCL6
GO:0001818∼negative regulation of cytokine production	2	0.046722	SLC11A1, BCL6
GO:0001775∼cell activation	3	0.049469	SLC11A1, BCL11B, BCL6
(B)			
GO:0043123∼positive regulation of I-kappaB kinase/NF-kappaB cascade	3	0.024598	CFLAR, TNFRSF10B, RHOC
GO:0043122∼regulation of I-kappaB kinase/NF-kappaB cascade	3	0.029497	CFLAR, TNFRSF10B, RHOC
GO:0009967∼positive regulation of signal transduction	4	0.037345	CFLAR, TNFRSF10B, ZAP70, RHOC
GO:0010647∼positive regulation of cell communication	4	0.04897	CFLAR, TNFRSF10B, ZAP70, RHOC
GO:0006915∼apoptosis	5	0.062711	CFLAR, TNFRSF10B, RAF1, MTP18, SOD1
GO:0006575∼cellular amino acid derivative metabolic process	3	0.065021	SLC22A4, ICMT, SOD1
GO:0012501∼programmed cell death	5	0.065518	CFLAR, TNFRSF10B, RAF1, MTP18, SOD1
GO:0010740∼positive regulation of protein kinase cascade	3	0.065708	CFLAR, TNFRSF10B, RHOC
GO:0006879∼cellular iron ion homeostasis	2	0.075126	HP, SOD1
GO:0055072∼iron ion homeostasis	2	0.086718	HP, SOD1
GO:0007242∼intracellular signaling cascade	7	0.090132	PDZD8, TNFRSF10B, ZAP70, RAF1, RHOC, SOD1, RAB27A
GO:0043065∼positive regulation of apoptosis	4	0.092511	CFLAR, TNFRSF10B, SOD1, RAB27A
GO:0043068∼positive regulation of programmed cell death	4	0.093994	CFLAR, TNFRSF10B, SOD1, RAB27A
GO:0010942∼positive regulation of cell death	4	0.094988	CFLAR, TNFRSF10B, SOD1, RAB27A
GO:0007010∼cytoskeleton organization	4	0.095487	SVIL, SSH2, RAF1, SOD1

### Construction and evaluation of the SVM classification model

**FIGURE 4:**
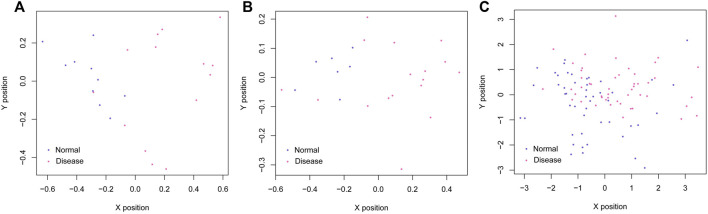
Scatterplots of the GSE61145 **(A)**, GSE60993 **(B),** and GSE34198 **(C)** datasets. The purple and red dots represent the normal and disease samples, respectively. The *X* and *Y* axes represent the position vector coordinates of the samples.

**FIGURE 5:**
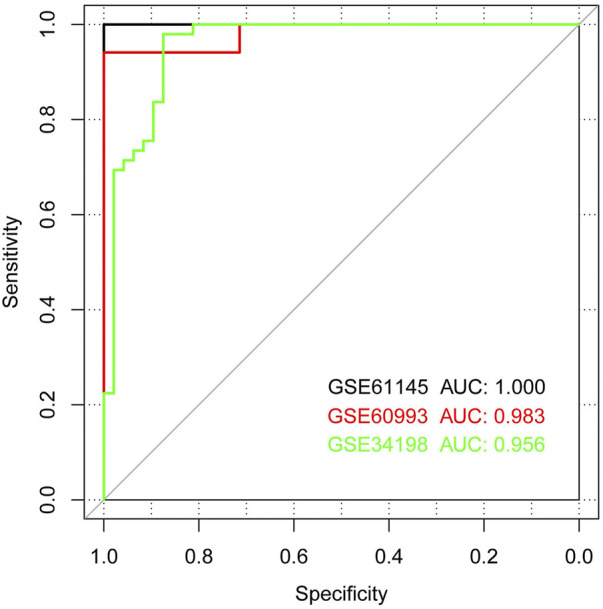
Receiver operating characteristic (ROC) curves showing classifier efficiency. The black, red, and green curves show the ROC curves of the GSE61145, GSE60993, and GSE34198 datasets, respectively.

Based on the SVM classification model, we removed genes that could not distinguish between the disease and control samples. Finally, seven genes were obtained: *ACOX1* (Acyl CoA oxidase 1), *ADCK2* (aarF domain containing kinase 2), *AFF3* (AF4/FMR2 family member 3), *BCL6* (B-cell lymphoma 6), *CEACAM8* (Carcinoembryonic antigen-related cell adhesion molecule 8), *CUGBP2* (CUG triplet repeat-binding protein 2) and *GPX7* (glutathione peroxidase 7). The SVM classification model of these seven genes could distinguish all samples in the GSE61145 dataset. The scatterplot of the GSE61145 dataset is shown in Figure 4A. The GSE60993 and GSE34198 datasets were then used as validation datasets to confirm the SVM classification model. As shown in Figure 4B, the SVM classification model correctly distinguished 23 (17 disease and 6 normal samples) of 24 samples in the GSE60993 dataset. Additionally, the scatterplot of the GSE34198 dataset indicated that the SVM classification model correctly distinguished 90 (48 disease and 42 normal samples) of the 97 samples (Figure 4C). The efficiency receiver operating characteristic (ROC) curves of the SVM classification model are shown in Figure 5 and the efficiency parameters of each dataset are listed in Table 3.

**TABLE 3:** Parameters for classifier performance.

Datasets	Num.Samples	Correct sample	Correct rate	Sensitivity	Specificity	PPV	NPV	Auroc
GSE61145	24	24	1.000	1.000	1.000	1.000	1.000	1.000
GSE60993	24	23	0.958	1.000	0.857	0.944	1.000	0.983
GSE34198	97	90	0.928	0.979	0.875	0.889	0.977	0.956

PPV, positive predictive value; NPV, net present value; AUROC, area under the receiver operating characteristic.

## Discussion

MI is a major cause of death and disability worldwide and has imposed burdens and impacted the health of the population (ErikssonP., 2014) While studies have focused on the mechanism and management of MI at the molecular level (Hak et al., 2000; Erikson et al., 2017; Wongsurawat, 2018; Yang et al., 2022), effective therapy is lacking. The present study screened 1,231 DEGs based on three microarray datasets. Based on WGCNA, the top three modules related to disease (black, pink, and red) were screened. Afterward, a total of 98 DEGs were screened from the top three modules to construct the gene co-expression network. The SVM classification model was also constructed and identified seven genes (including *ACOX1*, *BCL6*, *CEACAM8*, *CUGBP2,* and *GPX7*) that were closely associated with MI.

ACOX1 is the first enzyme in peroxisomal fatty acid β-oxidation. It is rate-limiting and plays a key role in fatty acid metabolism and fat deposition (Foraker et al., 2013). Both lipid abnormalities and chronic inflammation have crucial involvement in atherosclerosis initiation and progression (Bhagavan et al., 2003). Lutein plays a regulator role in gene expression and is involved in oxidative stress and the lipid metabolism of *ACOX1*, thereby mitigating atherosclerosis progression (Bruyninckx et al., 2008). In addition, *BCL6* is a transcriptional repressor required for mature B-cell germinal center (GC) formation and is also implicated in lymphomagenesis (Jiao et al., 2011; Vik et al., 2015). Increasing *Bcl6* expression reduces inflammatory responses and limits atherosclerosis (Han et al., 2015). Meanwhile, *CEACAM8* is a glycosylphosphatidylinositol-anchored membrane glycoprotein with a molecular weight of around 95 kDa (Basso et al., 2010). *CEACAM8* is also known as Cluster of Differentiation 66b (*CD66b*) and is expressed by neutrophils (Lasa et al., 2008; Kulbacki et al., 2010; Singer, 2013; Wei et al., 2015). Leucocyte activation is a crucial step in atherogenesis (Chudasama et al., 2011). The expression of leucocyte integrins, such as neutrophil and neutrophil *CD66b*, has been linked to atherosclerosis (Alipour et al., 2013). Furthermore, coronary artery disease (CAD) reflects generalized inflammation (Oostrom et al., 2004). Additionally, CUG triplet repeat-binding protein 2 (*CUGBP2*) plays a critical role in the apoptosis of breast cancer cells in response to genotoxic injury (Mukhopadhyay et al., 2004). The over-expression of miR-144 can decrease cardiomyocyte cell death by targeting *CUGBP2* (Alipour et al., 2013). miR-451 is also largely responsible for ischemic preconditioning-mediated cardioprotection, which also showed protective effects against simulated ischemia/reperfusion-induced cardiomyocyte death by *CUGBP2* regulation (Weiss et al., 2012; Chen et al., 2014; Feng et al., 2016). Subsequently, GPX7 is an endoplasmic reticulum (ER)-mitochondria protein that plays important and emerging functional roles in T-cell development (Higashi et al., 2013). Numerous clinical studies have found that hyperhomocysteinemia (HHcy) is an independent risk factor for cardiovascular diseases in humans (Chen et al., 2016). HHcy accelerates atherosclerosis by affecting the immuno-inflammatory response and repressing regulatory T-cell functions (Feng et al., 2016). Furthermore, the results of the gene co-expression network analysis in this study showed the co-expression of *BCL6*, *CEACAM8,* and *CUGBP2*. *ADCK2* and *AFF3* were also associated with MI in this study. However, evidence regarding their roles in MI is scarce. Thus, A*COX1*, *BCL6*, *CEACAM8*, *CUGBP2* and *GPX7* may play key roles in MI pathogenesis.

## Conclusion

Myocardial infarction is one of the most dangerous diseases worldwide. This study screened for genes associated with such diseases. We obtained gene expression datasets (GSE61145, GSE60993, and GSE34198) related to human MI. We searched microarray datasets involving human MI and then investigated the DEGs between MI and normal samples. The genes associated with MI were further screened by identifying the disease-associated modules to construct a gene co-expression network. *ACOX1*, *BCL6*, *CEACAM8*, *CUGBP2,* and *GPX7* might be key genes implicated in MI development. The MI-associated genes may provide targets for novel therapy for MI. As our findings were partially drawn by prediction, they require additional validation. However, this study has several limitations that should be addressed in future work. The SVM algorithm can be treated as a typical classification model in the field of bioinformatics and computational biology. Therefore, several classification algorithms, including random forest, neural network, and some deep learning algorithms, can be used to correct this issue. This study used the GSE61145 dataset to train the classification mode. Considering the generality of the classification model, more datasets should be trained. Future work should also utilize cross-validation methods.

## Data Availability

Publicly available datasets were analyzed in this study. The names of the repository/repositories and accession number(s) can be found in the article/supplementary material.
